# *In Vitro* Antimicrobial Activity of Taurolidine Against Isolates Associated with Catheter-Related Bloodstream Infections

**DOI:** 10.3390/antibiotics15080785

**Published:** 2026-08-14

**Authors:** Jared L. Crandon, Xing Tan, Paul R. Rhomberg, Mariana Castanheira, S. J. Ryan Arends

**Affiliations:** 1 CorMedix Therapeutics, Parsippany, NJ 07054, USA; 2 Element Iowa City (JMI Laboratories), North Liberty, IA 52317, USA

**Keywords:** taurolidine, multidrug resistance, catheter-related infection, infection control

## Abstract

Background: Catheter-related bloodstream infections are common in patients receiving hemodialysis through a central venous catheter. Taurolidine, a novel broad-spectrum antimicrobial catheter lock solution, is FDA-approved in combination with heparin for reducing the incidence of such infections. Methods: *In vitro* activity of taurolidine was assessed against 442 bacterial and 50 yeast clinical bloodstream infection isolates selected from the SENTRY Antimicrobial Surveillance Program and tested according to the Clinical and Laboratory Standards Institute methodology. Multidrug-resistant species and phenotypes were included. Results: Taurolidine was highly active against all isolates, with MIC_50_/MIC_90_ values of ≤1024/1024 μg/mL for most species. The highest MICs observed were among isolates of *Mycobacterium avium* complex and *Burkholderia cepacia* complex (MIC_90_, 2048 μg/mL) and *Candida albicans* (MIC_90_, 4096 μg/mL). All isolates were inhibited by 13,500 μg/mL of taurolidine, the concentration of the commercially available product. Conclusions: Taurolidine activity was very similar among Gram-positive, Gram-negative, and yeast clinical isolates, with the activity of taurolidine being unaffected by phenotypic resistance to other antimicrobials. Taken together with clinical efficacy data from clinical trials, this provides a theoretical basis for the idea that taurolidine-based lock solutions may be able to reduce the risk of catheter-related bloodstream infections from a wide variety of pathogens and resistance phenotypes.

## 1. Introduction

End-stage renal disease is the most severe form of chronic kidney disease and often requires renal replacement therapy, usually in the form of hemodialysis. In 2022, nearly 85% of patients starting hemodialysis in the United States required a central venous catheter, and 24.5% of all patients receiving hemodialysis required catheterization for therapy [1]. Among these patients, catheter-related bloodstream infections (CRBSIs) have an incidence of 1.1 to 5.5 episodes per 1000 catheter-days and up to half of patients experience a CRBSI within 6 months of catheter placement [2]. Antimicrobial catheter lock solutions are one option to help reduce the risk of CRBSI in patients receiving hemodialysis through a central venous catheter. 

Taurolidine is a broad-spectrum antimicrobial with a non-specific mechanism of action, exerting its effect through damage to microbial cell walls, preventing adherence of microorganisms to biological surfaces, and inhibiting select virulence factors (e.g., flagella and fimbriae) [3,4,5,6]. Taurolidine is the active component of DefenCath^®^ (13,500 μg/mL of taurolidine and 1000 units/mL of heparin) and represents the first FDA-approved catheter lock solution indicated to reduce the risk of CRBSI in adult patients with kidney failure receiving chronic hemodialysis through a central line [3]. Although used worldwide for many years, there is a paucity of recent data regarding the activity of taurolidine against microbial pathogens causing bloodstream infections. Additionally, individual isolates causing CRBSI from the LOCK IT-100 Phase 3 clinical trial were not available for susceptibility testing [7]. The objective of this study was to evaluate the *in vitro* antimicrobial activity of taurolidine against a set of recent clinical isolates representative of those recovered from the LOCK IT-100 trial and/or challenge isolates associated with CRBSI.

## 2. Results

Taurolidine exhibited broad antimicrobial activity against all isolates tested, which is summarized in Table 1. The taurolidine minimum inhibitory concentration (MIC) that inhibited 90% of isolates tested (MIC_90_) was ≤1024 μg/mL in 94.3% of all isolates, with 92.9% of all MICs within one 2-fold dilution of 512 μg/mL. Collectively, Gram-positive bacterial MIC_50/90_ values were 512/512 μg/mL and Gram-negative bacterial MIC_50/90_ values were 512/1024 μg/mL. Higher MIC_90_ values were observed in *Mycobacterium avium* complex and *Burkholderia cepacia* complex (MIC_90_, 2048 μg/mL), as well as *Candida albicans* (MIC_90_, 4096 μg/mL). Taurolidine activity against *C. glabrata* and *C. parapsilosis* was similar to that against bacterial isolates, with MIC_50/90_ values of 512/512 μg/mL and 256/512 μg/mL, respectively. Among the non-tuberculous Mycobacteria (NTM) tested, greater activity was seen against *M. abscessus* (MIC_50/90_ 512/1024 μg/mL) than *M. avium* complex (MIC_50/90_ 2048/2048 μg/mL). 

## 3. Discussion

To our knowledge, this is the most recent assessment of the activity of taurolidine against modern clinical isolates causing bloodstream infections. As individual isolates recovered during the Phase 3 clinical trial were not available for testing, this study evaluated the *in vitro* antimicrobial activity of taurolidine against a set of recent clinical isolates representative of those recovered from the LOCK IT-100 trial (Table 2) and challenge isolates commonly associated with CRBSI or uncommon but difficult-to-treat causes of CRBSI [7,8]. In the LOCK IT-100 trial comparing the time to CRBSI of the taurolidine/heparin catheter lock solution to that of heparin alone, 43 isolates were recovered from 41 cases of CRBSI (9 and 32 cases in the taurolidine/heparin and heparin alone arms, respectively [*p* = 0.0006]) [7]. *S. aureus* was the most common pathogen in both groups, representing 36.6% of CRBSI cases, with other notable pathogens including coagulase-negative Staphylococci (CoNS), *E. faecalis*, *Enterobacter cloacae*, and *K. pneumoniae*. 

In the present study, *S. aureus*, which is the organism most commonly associated with CRBSI in patients undergoing hemodialysis [9,10], was consistently inhibited by taurolidine at concentrations more than 20-fold lower than those used clinically (MIC_50/90_ 512/512 μg/mL), and β-lactam resistance had no impact on taurolidine activity. Overall, taurolidine activity was consistent across microbial genera and activity was maintained regardless of phenotypic resistance to typical antibiotics (e.g., methicillin-resistant *Staphylococcus aureus* [MRSA], vancomycin-resistant *Enterococcus* [VRE], multidrug-resistant [MDR] *Pseudomonas aeruginosa*). No formal susceptibility breakpoints are available for taurolidine; however, subpopulations with increased MICs appear to be rare, and the mutation frequency has been reported to be <10^−9^ at 2× and 4× the MIC [11]. Importantly, microbiologic resistance above clinical concentrations leading to clinical failure has never been reported among many clinical trials [12,13,14,15,16]. One study specifically assessed the potential microbial adaptation among isolates recovered from patients receiving parenteral nutrition who developed CRBSI despite using taurolidine lock therapy. Among the 27 isolates recovered, the MIC values were comparable to those reported here and elsewhere, suggesting a lack of adaptation even among patients who develop CRBSI [12]. 

As the available formulation of taurolidine/heparin catheter lock solution contains 13,500 μg/mL of taurolidine, reliable inhibition of the isolates exhibiting higher MICs could be expected, such as *B. cepacia* complex, *M. avium* complex, and *C. albicans*. While CRBSIs due to these isolates are less common, broad-spectrum activity against such difficult-to-treat isolates may be desired in certain situations or at-risk populations [8,17]. The growing global burden of antimicrobial resistance, especially among Gram-negative bacteria such as *A. baumannii*, and risks of candidemia in patients with indwelling central-venous access devices, emphasizes the importance of challenge isolates included in this report [18,19,20,21,22,23]. 

These findings are consistent with previous *in vitro* studies evaluating taurolidine activity against various Gram-positive, Gram-negative, and *Candida* species [11,24]. Other notable pathogens that taurolidine has demonstrated *in vitro* activity against include *C. auris*, *Rhizopus* species, and *Aspergillus fumigatus* [25,26,27]. All isolates of these three pathogens were reported to be completely inhibited in planktonic growth by taurolidine concentrations ≤ 1024 μg/mL. In addition, clinical studies and reports have assessed the potential of taurolidine to prevent many types of prosthetic-device-related infections other than CRBSI in hemodialysis, such as with parenteral nutrition [28,29,30,31,32], peritoneal dialysis [33,34], malignancy [35,36,37,38,39], and cardiac implantable electronic devices [40]. Results are generally favorable, with several meta-analyses reporting efficacy in prevention of CRBSI and central-line-associated bloodstream infection [14,15,16]. Although less frequently implicated, *B. cepacia* complex and NTM have also been reported in association with CRBSIs among patients receiving hemodialysis and other populations requiring central venous access. 

*B. cepacia* complex is an important nosocomial pathogen. In a US case–control study of ICU patients without cystic fibrosis, renal failure requiring dialysis and the presence of a central line were among the factors independently associated with development of *B. cepacia* complex bacteremia [41]. A multicenter Indian study showed *B. cepacia* complex was isolated from 5 to 10% of the central-line-associated bloodstream infection isolates in adult ICUs [23]. In a small Taiwanese study, *B. cepacia* complex was isolated in two of eight cases of hemodialysis CRBSI prior to the implementation of a novel infection prevention protocol [42]. *B. cepacia* complex has also been associated with several outbreaks among hemodialysis patients associated with facility water and plumbing issues, in addition to reusing certain forms of hemodialyzers, emphasizing the need for a multimodal approach to infection prevention [43,44,45,46]. Conversely, NTM are more prone to infecting immunocompromised hosts, with catheter-related infections being rare but challenging [47,48]. The most common fast-growing NTM for CRBSI is *M. abscessus*, with *M. avium* complex being the most common slow-growing NTM [49]. NTM bloodstream infections occur primarily in patients with intravascular catheters and underlying immunocompromising conditions, particularly malignancy and hematopoietic stem cell transplantation [48,50,51,52]. Rapidly growing Mycobacteria account for most reported NTM bloodstream isolates in these populations, with *M. abscessus* or *M. abscessus* complexes identified among causative organisms across multiple series. In the dialysis population, *M. abscessus* has been reported to be a cause of hemodialysis catheter-associated infection, and was the most frequently identified pathogen in a systematic review of peritoneal dialysis-related NTM catheter infections and peritonitis [53,54,55]. While exceptionally rare among most patients, catheter-related infections due to *M. avium* complex are reported more frequently among peritoneal dialysis patients than other forms of long-term indwelling catheters [55,56]. 

This study has limitations. Firstly, the *in vitro* activity of taurolidine does not necessarily translate to clinical efficacy. However, taurolidine/heparin lock solution was shown to reduce the risk of CRBSI by 71% compared to heparin alone and is FDA-approved to reduce the incidence of CRBSI in adults receiving chronic hemodialysis through a central venous catheter [3]. Secondly, the isolates in the current study are not those isolated from the LOCK IT-100 clinical trial, limiting direct interpretation of activity to efficacy. Most of the bacterial isolates included in this study were chosen to be representative of those isolates causing CRBSI in the clinical trial, demonstrating potent antimicrobial activity among a similar panel of clinical isolates to those observed. Third, this study did not complete further *in vitro* analysis of the activity of taurolidine on the isolates studied, such as static concentration time-kill experiments, biofilm eradication, or assessing the effect of serum proteins on activity. Other reports have demonstrated the concentration-dependent activity of taurolidine, with a minimum microbicidal concentration of approximately 1–4× the MIC [11,57,58,59]. In one study assessing Gram-positive bacteria, Gram-negative bacteria, and *Candida albicans*, undiluted taurolidine (13,500 μg/mL) caused eradication of planktonic cells, prevention of catheter colonization, and a median reduction in organisms in biofilms by 4.8 log_10_ colony-forming units per cm^2^ in 24 h [57]. Another study using 65% bovine serum showed modestly decreased activity of taurolidine at 2000 μg/mL, and in the presence of 65% defibrinated human blood, bactericidality often required overnight incubation [59]. These studies emphasize the importance of high taurolidine concentrations when used as a catheter lock solution. Finally, of the isolates assessed here, relatively few with resistant phenotypes were included; therefore, these results must be interpreted with caution. The antimicrobial activity data reported here is supported by decades of previous reports which have shown *in vitro* taurolidine activity consistent with the MIC_50/90_ of this work, including less common causes of CRBSI in hemodialysis, such as Enterococci, *A. baumannii*, *B. cepacia* complex, *S. maltophilia*, and *Candida* species [11,24]. While one isolate of *S. maltophilia* was seen in the control group but not the taurolidine group of the LOCK IT-100 trial, these challenge isolates have not been studied in the context of taurolidine reducing the risk of CRBSI in hemodialysis, and further clinical studies are warranted to confirm the *in vitro* findings. 

## 4. Materials and Methods

*In vitro* activity of taurolidine was assessed against 442 bacterial and 50 yeast isolates selected from the SENTRY Antimicrobial Surveillance Program. Given the breadth of the SENTRY Antimicrobial Surveillance Program, a convenience sample of randomly selected isolates was taken to be representative of those isolated in the LOCK IT-100 trial (Table 2), in addition to select challenge isolates with more resistant species and phenotypes. All isolates were collected from the bloodstreams of U.S. patients between 2018 and 2023 and included resistant phenotypes of common pathogens causing CRBSI (e.g., MRSA, VRE, MDR *P. aeruginosa*), as well as uncommon pathogens known for being especially difficult to treat (e.g., NTM, *S. maltophilia*, *B. cepacia* complex). Taurolidine activity against *Candida auris* has been reported previously [25]. 

MIC testing was completed in triplicate, with modal MIC being reported according to broth microdilution guidelines of the Clinical and Laboratory Standards Institute, including recommended quality control strains and ranges [60,61,62]. Taurolidine was solvated and diluted in DMSO prior to its addition to growth media at appropriate concentrations. Test panels were frozen and stored at −80 °C until use. Cation-adjusted Mueller–Hinton broth was used for testing bacterial isolates, which was supplemented with 2.5–5% lysed horse blood for Streptococci or 5% oleic acid, albumin, dextrose, and catalase when testing *M. avium* complex. Fungal isolates were tested using RPMI 1640 broth buffered with MOPS and 0.2% (*w*/*v*) glucose. As interpretive criteria have not been established for taurolidine susceptibility, MICs were read at 100% growth inhibition.

## 5. Conclusions

Taurolidine activity was very similar among a large collection of Gram-positive, Gram-negative, and yeast organisms, and activity was unaffected by phenotypic resistance to other antimicrobials. MIC_90_ values for all species/groups were ≤1024 μg/mL, more than 10-fold lower than clinical concentrations, except for *M. avium* complex and *B. cepacia* complex (MIC_90_, 2048 μg/mL) and *C. albicans* (MIC_90_, 4096 μg/mL) where slightly higher MIC_90_ values were observed. Based on these data, catheter lock solutions containing the broad-spectrum antimicrobial taurolidine at 13,500 μg/mL may have the potential to reduce the risk of CRBSI caused by a variety of species, including those observed in the recent LOCK IT-100 clinical trial and other bloodstream pathogens. Further clinical studies are required to validate these findings.

## Figures and Tables

**Table 1:** Distributions of taurolidine MIC values against various species/groups.

	No. of Isolates Inhibited at a Taurolidine MIC (μg/mL) of:								Taurolidine	
Organism (No. Isolates)	≤32	64	128	256	512	1024	2048	4096	MIC_50_	MIC_90_
*S. aureus* (76)				1	75				512	512
MSSA (37)					37				512	512
MRSA (39)				1	38				512	512
CoNS (52) ^a^				10	38	4			512	512
*S. epidermidis* (36)					32	4			512	1024
MSCoNS (21)				7	14				512	512
MRCoNS (31)				3	24	4			512	1024
*Enterococcus* species (48)				6	40	2			512	512
*E. faecalis* (38)				1	35	2			512	512
*E. faecium* (10)				5	5				256	512
VRE (6) ^b^				3	3				256	ND
Viridans group streptococci (18) ^c^		1	2	5	10				512	512
Non-tuberculous *Mycobacteria* (21)					7	7	7		1024	2048
*M. avium* complex (11) ^d^						4	7		2048	2048
*M. abscessus* (10)					7	3			512	1024
Enterobacterales (137)			1	22	106	8			512	512
*E. coli* (44)				1	43				512	512
*K. pneumoniae* (43)				2	38	3			512	512
*P. mirabilis* (10)				10					256	256
*E. cloacae* sc (10)					6	4			512	1024
*Citrobacter* species (10)				9	1				256	256
*S. marcescens* (20)			1		18	1			512	512
MDR Enterobacterales (20) ^e^				1	17	2			512	512
*P. aeruginosa* (45)					20	23	2		1024	1024
MDR *P. aeruginosa* (10)					8	2			512	1024
*S. maltophilia* (15)				12	2	1			256	512
*A. baumannii-calcoaceticus* sc (15)				2	13				512	512
MDR *A. baumannii* sc (6)				1	5				512	ND
*B. cepacia* sc (15)				10	1	2	2		256	2048
*C. albicans* (17)							5	12	4096	4096
*C. glabrata* (17)				3	13	1			512	512
*C. parapsilosis* (16)			3	9	4				256	512

MSSA, methicillin-susceptible *S. aureus*; MRSA, methicillin-resistant *S. aureus*; CoNS, coagulase-negative Staphylococci; MSCoNS, methicillin-susceptible coagulase-negative Staphylococci; MRCoNS, methicillin-resistant coagulase-negative Staphylococci; ND, no data (fewer than 10 isolates precludes calculating an MIC_90_); VRE, vancomycin-resistant Enterococci; MDR, multidrug-resistant. ^a^ *Staphylococcus capitis* (2), *S. epidermidis* (36), *S. haemolyticus* (2), *S. lugdunensis* (10), and *S. saprophyticus* (2). ^b^ *Enterococcus faecium* (6). ^c^ *Streptococcus anginosus* group (2), *S. bovis* group (5), *S. gallolyticus* (2), *S. mitis* group (2), *S. salivarius* group (2), and *S. sanguinis* (5). ^d^ *Mycobacterium avium* (6) and *Mycobacterium intracellulare* (5). ^e^ *Enterobacter cloacae* species complex (2), *Escherichia coli* (9), and *Klebsiella pneumoniae* (9).

**Table 2:** Pathogens recovered from CRBSI during LOCK IT-100.

	Taurolidine/Heparin		Heparin	
Organism	n	%	n	%
Total	9		34 ^a^	
Gram-Positive	8	88.9%	20	58.8%
*S. aureus*	7	77.8%	8	23.5%
MSSA	3	33.3%	3	8.8%
MRSA	4	44.4%	5	14.7%
*S. epidermidis*	1	11.1%	4	11.8%
*S. lugdunensis*			1	2.9%
*S. sanguinis*			2	5.9%
*S. bovis*			1	2.9%
Viridans streptococci			1	2.9%
*E. faecalis*			3	8.8%
Gram-Negative	1	11.1%	14	41.2%
*C. koseri*			2	5.9%
*E. cloacae*			4	11.8%
*K. aerogenes* ^b^			1	2.9%
*K. pneumoniae*	1	11.1%	2	5.9%
*S. maltophilia*			1	2.9%
*S. marcescens*			3	8.8%
*P. aeruginosa*			1	2.9%

^a^ Polymicrobial: *P. aeruginosa* and *S. epidermidis*; *S. epidermidis* and Viridans streptococcus. ^b^ Previously *Enterobacter aerogenes*.

## Data Availability

The original contributions presented in this study are included in the article. Further inquiries can be directed to the corresponding author.
